# Supplementary material for: Analysis of Characteristics of Bovine-Derived Non-Enterotoxigenic Bacteroides fragilis and Validation of Potential Probiotic Effects
Source: Microorganisms. 2024 Nov 14;12(11):2319. doi: 10.3390/microorganisms12112319 (PMC11596406; doi:10.3390/microorganisms12112319)
Supplement: Supplementary file 1 [file microorganisms-12-02319-s001.zip › microorganisms-3248999-supplementary.pdf]

Journal name:

Microorganisms

Manuscript Title:

Analysis of characteristics of bovine-derived non-enterotoxigenic *Bacteroides fragilis* and validation of potential probiotic effects

Kangkang Guo, Ph.D. Professor

College of Veterinary Medicine, Northwest A&F University

No. 22 Xinong Road, Yangling, Shaanxi 712100, China.

Tel: +86-029-87092040

Email: guokk2007@nwsuaf.edu.cn

Table S1. The total number of samples with diarrhea stools and normal stools.

Farm	Number of calves with diarrhea	Number of normal calves
A. Shangle	7	5
B. Wuli	6	6
Total	13	11

Table S2. Contig statistics after assembly.

Sample name	Total number (>500bp)	Total length (bp)	N50 length (bp)	N90 length (bp)	Max length (bp)	Min length (bp)	Sequence GC%
BF-1153	68	5,469,519	274,500	106,978	1,095,986	514	43.42

Table S3. The information of the predicted genomes.

Sample name	K-mer	K-mer Number	K-mer Depth	Genome Size (Mb)	Revised Size (Mb)	Heterozygous Rate (%)	Repeat Rate (%)
BF-1153	15	294,029,054	51.52	5.71	5.56	0.04	17.26

Note: K-mer Number, the number of k-mers extracted from reads for statistics; K-mer Depth, statistical k-mer average depth; Genome Sze, estimated genome size; Revised Size, corrected estimated genome size; Heterozygous Rate, estimated percentage of genomic heterozygosity; Repeat Rate, estimated genome repeat ratio.

Table S4. Predicted coding genes.

Sample ID	Genome size (bp)	Gene number (#)	Gene total length (bp)	Gene average length (bp)	Gene length/Genome (%)
BF-1153	5,469,519	4,730	4,833,822	1,022	88.38

Table S5. Scattered repeat sequence results.

Sample ID	Type	Number(#)	Total length(bp)	In Genome(%)	Average length(bp)
BF-1153	Unknown	1	73	0.0013	73
BF-1153	RC	4	322	0.0059	81
BF-1153	SINE	6	346	0.0063	58
BF-1153	LINE	49	2748	0.0502	56
BF-1153	DNA	54	3085	0.0564	59
BF-1153	LTR	182	11036	0.2018	61
BF-1153	Total	296	17610	0.3174	60

Note: From left to right are the types of scattered repeats, number of repeats, total length of repeats, percentage of repeats in the genome, and average length of the repeats. LTR, long terminal repeats; DNA, DNA transposon; LINE, long scattered repetitive sequences; SINE, short scattered repetitive sequences; RC, rolling circle.

Table S6. Tandem repeat sequence results.

Sample ID	Type	Number(#)	Repeat size(bp)	Total length(bp)	In Genome(%)
BF-1153	Micro DNA	0	0-0	0	0
BF-1153	Mini DNA	153	10-60	7667	0.1402
BF-1153	TR	160	7-207	9024	0.1650

Note: From left to right are the type of tandem repeats, number of repeats, length range of repeats, total length of repeats, and percentage of repeats in the genome. TR, tandem repeats; Mini DNA, Minisatellite DNA; Micro DNA, Microsatellite DNA.

Table S7. The statistical results of ncRNA after redundancy removal.

Sample ID	Type	Number(#)	Average length(bp)	Total length(bp)
BF-1153	tRNA	71	77	5791
BF-1153	5s(denovo)	7	110	770
BF-1153	16s(denovo)	1	1517	1517
BF-1153	23s(denovo)	1	2886	2886
BF-1153	sRNA	0	0	0

Note: From left to right are the types of ncRNAs, number of ncRNAs, average length of ncRNAs, and total length of ncRNAs.

Table S8. The prediction results of Prophages.

Sample ID	Prophage number (#)	Total length (bp)	Average length (bp)
BF-1153	12	612825	51068.8

Note: From left to right are the sample ID, number of prophages, total length of the prophages, and average length of the prophages.

Table S9. The prediction results of CRISPR.

Sample ID	CRISPR number (#)	Total length (bp)	Average length (bp)
BF-1153	3	1880	626.667

Note: From left to right are the sample ID, the total number of CRISPR predicted, the total length of CRISPR, and the average length of CRISPR.
